# Supplementary material for: Diverse Interactions of Sterols with Amyloid Precursor Protein Transmembrane Domain Can Shift Distribution Between Alternative Amyloid-β Production Cascades in Manner Dependent on Local Lipid Environment
Source: Int J Mol Sci. 2025 Jan 10;26(2):553. doi: 10.3390/ijms26020553 (PMC11764862; doi:10.3390/ijms26020553)
Supplement: Supplementary file 1 [file ijms-26-00553-s001.zip › ijms-3370773-supplementary.doc]

**Supplementary Data**

**Diverse Interactions of Sterols with Amyloid Precursor Protein Transmembrane Domain Can Shift Distribution Between Alternative Amyloid-β Production Cascades in Manner Dependent on Local Lipid Environment**

## **Pavel E. Volynsky^1^**^†^**, Anatoly S. Urban^1^**^†^**, Konstantin V. Pavlov^2^, Yaroslav V. Bershatsky^1,2^, Olga V. Bocharova^1^, Anastasia K. Kryuchkova^2^, Veronika V. Zlobina^1,2^, Alina A. Gavrilenkova^1,2^, Sofya M. Dolotova^1,2^, Anna V. Kamynina^1,2^, Olga T. Zangieva^3^, Amir Taldaev^2,4^, Oleg V. Batishchev^5^, Ivan S. Okhrimenko^2^, Tatiana V. Rakitina^1*^, Roman G. Efremov^1,6^, Eduard V. Bocharov^1,2*^**

*^1^ Shemyakin–Ovchinnikov Institute of Bioorganic Chemistry RAS, Moscow, 117997, Russian Federation*

*^2^ Moscow Center of Advanced Studies, Moscow, 123592, Russia*

*^3^ Pirogov National Medical and Surgical Center, Moscow, 105203, Russia*

*^4^ Institute of Biomedical Chemistry, Moscow, 119121, Russia*

*^5^ Frumkin Institute of Physical Chemistry and Electrochemistry RAS, Moscow, 119071, Russia*

*^6^ Department of Applied Mathematics, National Research University Higher School of Economics, Moscow 101000, Russia*

^†^ These authors have contributed equally to this work.

**^*^ Correspondence:** taniarakitina@yahoo.com, bon@nmr.ru

**Figure S1.** **Four independent MD simulations (A-D) of the sterol interactions with the APPjmtm peptide in the explicit POPC bilayer: site #1.** The doxyl-cholestane molecule was initially constrained (for 20 ns) in the identified sterol-binding site #1, corresponding to a characteristic pattern near the APP JM helix, and then released for 100-ns MD relaxation. *From left to right*, the maps of the direct contacts (with 4 Å distance cut-off between heavy atoms) of APPjmtm residues with the sterol molecule (with all heavy atoms or with polar headgroup only) and the maps of the protein secondary structure (color coding corresponding to Figure 3C) evolution during the unconstrained MD simulations.

**Figure S2.** **Four independent MD simulations (A-D) of the sterol interactions with the APPjmtm peptide in the explicit POPC bilayer: site #2.** The doxyl-cholestane molecule was initially constrained (for 20 ns) in the identified sterol-binding site #2, corresponding to a characteristic pattern near the N-terminal G^700^xxxG^704^ motif of the APP TM helix, and then released for 100-ns MD relaxation. *From left to right*, the maps of the direct contacts (with 4 Å distance cut-off between heavy atoms) of APPjmtm residues with the sterol molecule (with all heavy atoms or with polar headgroup only) and the maps of the protein secondary structure evolution during the unconstrained MD simulations.

**Figure S3.** **Four independent MD simulations (A-D) of the sterol interactions with the APPjmtm peptide in the explicit POPC bilayer: site #3.** The doxyl-cholestane molecule was initially constrained (for 20 ns) in the identified sterol-binding site #3, corresponding to a characteristic pattern near the G^709^xxxA^713^ motif in the central part of the APP TM helix, and then released for 100-ns MD relaxation. *From left to right*, the maps of the direct contacts (with 4 Å distance cut-off between heavy atoms) of APPjmtm residues with the sterol molecule (with all heavy atoms or with polar headgroup only) and the maps of the protein secondary structure evolution during the unconstrained MD simulations.
